# Editorial: Breast milk: From nutrition to immunological action

**DOI:** 10.3389/fimmu.2022.1036008

**Published:** 2022-10-18

**Authors:** Adenilda Cristina Honorio-França, Luis Carlos de Abreu, Khalifa Elmusharaf, Eduardo Siqueira, Eduardo Luzía França

**Affiliations:** ^1^ Institute of Biological and Health Science, Federal University of Mato Grosso, Barra do Garças, Brazil; ^2^ Department of Integrated Health Education, Federal University of Espirito Santo, Vitória, Brazil; ^3^ School of Medicine, University of Limerick, Limerick, Ireland; ^4^ Department of Urban Planning and Community Health, University of Massachusetts Boston, Boston, MA, United States

**Keywords:** breastfeeding, maternal diseases, immune cells, nutrition, bioactives factors

Human milk has adequate amounts of macro and micronutrients and bioactive components that help in the maturation of the immune system and the development of the neonate (1). The maternal health status can directly interfere with the composition of breast milk and the pathologies existing before the gestational period (2, 3). Breast milk, in addition to its nutritional importance, is rich in defense factors that work as an immunological complement (1, 2).

Changes in the immune system and maternal metabolism probably affect the development of the neonate’s immune system, increasing the risk of infection. However, due to the intense interaction between mother and child during breastfeeding, this risk can be minimized by protective mechanisms involving bioactive factors with immunomodulatory action. Nevertheless, there are still several gaps regarding the mechanisms of action and the benefits of the immunological components of human milk, both for children and mothers.

We, therefore, decided to encourage researchers working on human milk to continue contributing to a better understanding of the nutritional and immunological aspects of human milk associated with maternal diseases and infections, highlighting gaps in the literature, advancing this scientific knowledge, and resulting in an improvement in quality of life for mothers and their children. Therefore, presenting this research topic with 8 manuscripts and 69 authors is a pleasure. In the following sections, we highlight the relevance of these publications.

Early nutrition through breastfeeding is an important environmental input that can induce lifelong effects on metabolism, growth, and important pathological processes such as obesity, diabetes mellitus, and breast cancer (2, 4). In this Research Topic, Lokossou et al. comprehensively review the evidence on the mechanisms underlying the roles of breast milk components, in particular immune cells, that are involved in infant protection against infections and diseases related to the immune system. Further, they highlight the importance of non-communicable diseases such as breast cancer, overweight, obesity, and diabetes.

The contributors also considered concern about the impact of non-communicable maternal diseases, such as diabetes, on breastfeeding. For example, Avellar et al. show that colostrum composition is modified in mothers with gestational diabetes mellitus and that certain cytokines and growth factors have their concentration altered compared to healthy mothers, which reinforces the idea that alterations in the maternal metabolism lead to immunological changes in colostrum.

The soluble and cellular components of human breast milk vary according to the period of milk maturation and the time of day (5). The nutritional and immunological composition also changes depending on the diet and diseases experienced by the mother. In this regard, Hicks et al. report the presence of non-coding RNAs (ncRNAs) in breast milk that confers metabolic and immunological effects on the infant. MicroRNAs vary depending on the maturity of the milk and maternal diet. MicroRNAs play an important role in maternal breast function and contribute to infant immune development and metabolism. Furthermore, Chen et al. provide comprehensive data on the presence of sialic acid in human milk, which is dependent on the time of lactation. The increased sialic acid concentration was also observed in the milk of preterm mothers, and sialic acid content was associated with maternal age, pre-gestational Body Mass Index, and type of delivery.

In addition to the nutritional and immunological components of human milk, Ganeshalingam et al. provide a concise review of the role of lipidomics in assessing breast milk and the functional lipid composition by which breast milk affects the health of the newborn. They point out that lipids not only provide energy but also play some additional roles, such as aiding in the development of the immune and neurological systems and regulating metabolism. Furthermore, He et al. emphasize that oral administration of glycerol monolaurate can delay the occurrence of DSS-induced colitis, regulating the immune balance, mainly cytokines and immune cells, protecting the intestinal mucosal barrier, and improving the intestinal microbiota. This study provides insights into glycerol monolaurate biological function and therapeutic potential in treating inflammatory bowel disease. Furthermore, Andrade et al. address the efficacy of a mixture of probiotics (*Lactobacillus* and *Bifidobacterium*) in children and adolescents with atopic dermatitis (AD) and the effects on sensitization, inflammation, and immune tolerance. They also emphasize that the microbiological profile of the gastrointestinal tract is higher in infants exclusively breastfed with human milk.

Finally, the COVID-19 pandemic was a major event that massively affected the entire world. In this Research Topic, Garib et al. present an experimental validation for obtaining milk with neutralizing antibodies against SARS-CoV-2 and show that the pasteurization of retained milk specifically neutralizes the activity of the virus, which can confer passive immunity against the infection by coronavirus as a complementary approach to vaccination.

Therefore, we invite the readers to analyze in detail the findings of these articles and discuss, criticize, and replicate the results. This invitation extends to researchers working on breastfeeding who could contribute to scientific advances in the area and encourage mothers to continue to breastfeed their children since human milk is an excellent source of food for newborns and can reduce high rates of maternal and infant complications.

## Author contributions

All authors listed have made a substantial, direct, and intellectual contribution to the work and approved it for publication.

## Funding

The authors would like to thank the Fundação de Amparo à Pesquisa do Estado de São Paulo (FAPESP- process number 2019/25112-2) and the Federal University of Mato Grosso - Publication of Support to Research (2021).

## Conflict of interest

The authors declare that the research was conducted in the absence of any commercial or financial relationships that could be construed as a potential conflict of interest.

## Publisher’s note

All claims expressed in this article are solely those of the authors and do not necessarily represent those of their affiliated organizations, or those of the publisher, the editors and the reviewers. Any product that may be evaluated in this article, or claim that may be made by its manufacturer, is not guaranteed or endorsed by the publisher.
